# Molecular pathological expression in malignant gliomas resected by fluorescein sodium-guiding under the YELLOW 560 nm surgical microscope filter

**DOI:** 10.1186/s12957-018-1495-2

**Published:** 2018-10-01

**Authors:** Ningning Zhang, Zhende Shang, Zhigang Wang, Xianbing Meng, Zheng Li, Hailong Tian, Dezhang Huang, Xin Yin, Bin Zheng, Xinhua Zhang

**Affiliations:** 1 Department of Neurosurgery, Shandong University Qilu Hospital, Qingdao, Shandong China; 2 Department of Neurosurgery, Affiliated Hospital of Taishan Medical University, Tai An, Shandong China

**Keywords:** Fluorescein sodium, YELLOW 560 nm, Malignant glioma, GFAP, Ki-67

## Abstract

**Background:**

This study aimed to analyze the relationship between molecular pathologic expression of GFAP and Ki-67 and fluorescence levels, and to provide molecular pathological basis for the removal of malignant gliomas (MG) by Fluorescein Sodium (FLS) navigation under the YELLOW 560 nm surgical microscope filter.

**Methods:**

A retrospective analysis of clinical data of 18 MG cases confirmed by the postoperative pathology was performed. All cases were resected by FLS guiding under the YELLOW 560 nm filter. Hematoxylin-eosin (HE) staining, molecular pathology markers GFAP, and Ki-67 immunohistochemical staining of the specimens were performed. The relationship between fluorescence staining levels and GFAP positive rate, Ki-67 proliferation index, and WHO grades was studied.

**Results:**

There were 69 pathological specimens with fluorescence levels of “bright” fluorescence (*n* = 32), “low” fluorescence (*n* = 18), and “no” fluorescence (*n* = 19). Immunohistochemical staining showed GFAP-positive expression in both tumor cells and normal glial cells. The staining levels of the specimens in the fluorescence regions were higher than that in the non-fluorescence regions. GFAP expression was positive in 61 specimens and negative in 8 specimens. Comparison of Ki-67 proliferation index using chi-square test showed different fluorescence levels had different Ki-67 proliferation indexes (*χ*^*2*^ = 14.678, *p* = 0.005). With high proliferation index of specimens, fluorescence level was brighter. WHO grade had no correlation with fluorescence levels (*χ*^*2*^ = 3.531, *p* = 0.171).

**Conclusion:**

FLS-guided resection of MG is safe and effective. In the boundary area of MG, fluorescence levels and Ki-67 proliferation index showed correlation. FLS-guided resection achieved the function of “reducing tumor cell,” thus reducing the proliferation index in the lesion area.

## Background

Malignant gliomas (MG) are graded as WHO grade III or IV gliomas. Glioblastoma is the most common malignant primary tumor of the skull. Adult glioblastoma accounted for 15.4% of all primary brain tumors and 45.6% of primary malignant tumors [1]. The treatment of glioblastoma remained difficult, demonstrating poor prognosis, high recurrence rate, high mortality rate, and low cure rate. Recently, several treatment strategies have been developed, including microsurgical treatment, chemotherapy, radiotherapy, and other modern therapeutic models, but the prognosis improvement still remained difficult. The increased total resection rate can improve the progression-free survival (PFS) and the overall survival (OS) rate of the patients with MG [2, 3]. The key treatment for this type of tumor is to maximize safe resection of the tumor. The pathological occurrence of glioma involves multiple-sources and is highly malignant and invasive. The boundary between the tumor and normal brain tissue is indistinctive and remains difficult for the complete removal of tumor cells. Several experimental studies both in vivo and in vitro have confirmed [4–6] that even 2 cm residual area outside glioblastoma can cause tumor recurrence and poor prognosis. Many surgical techniques have been developed, including neuronavigation, intraoperative ultrasound, electrophysiological monitoring during operation, magnetic resonance during surgery, fluorescence guided surgery, and so on, to improve the total resection rate and improve the patients’ disease progressive-free and overall survival rate. The technique of Fluorescein Sodium (FLS) navigation for the resection of MG is used to mark the tumor during operation and assist the doctors for removing tumor. Although 5-acetyl propionic acid(5-ALA) was also useful in guided resection of glioblastoma, but it had much difficulty in clinical popularization because of many disadvantages, such as not approved by the Committee on Food and Drug Administration, photo-induced toxicity, expensive equipment, and complicated application process. In 2013, K.M. Schebesch et al. [7] reported the use of FLS under YELLOW 560 nm to resect intracranial tumors in 35 cases. This study supported the use of FLS navigation resection as a safe and effective method for MG. However, there is a lack of quantitative study on the fluorescence intensity of FLS navigation of MG as these are different from other malignant tumors, and MG rarely metastasize to other different organs. These in turn often lead to death by invading the underlying brain tissues and resistance to modern treatment strategies.

Histological grading of glioma is based on modern histological features, including necrosis, cell nuclear polymorphism, nuclear fission ability, angiogenesis, and so on. The molecular pathology and immunohistochemistry were applied clinically to investigate individually and monitor the patients from the gene and protein levels. Immunohistochemical study of molecular markers has been more accurate and added practical value in the pathological diagnosis and prognostic judgment. Many molecular markers are used for guiding diagnosis, differential diagnosis, tumor malignancy, treatment guidance, and prognosis evaluation.

In this study, glial fibrillary acidic protein (GFAP) and proliferation-related protein Ki67 were selected as the classic markers of glioma, and the different fluorescence level specimens were analyzed by immunohistochemical pathology and operation. Our study aimed to further investigate the differential expression of molecular pathology of MG under different fluorescence levels to provide a basis for the use of FLS in the navigation of the MG cells and more effective identification of the boundary between the tumor and the brain. We hope to better guide the application of FLS navigation, protect the normal brain tissue, improving the surgical resection rate and prognosis.

## Methods

### Data collection

Retrospective analysis of the 18 cases of pathologically confirmed MG resected by FLS guiding under the YELLOW 560 nm filter from the neurosurgery department of Shandong University Qilu Hospital (Qingdao) between January 2014 and December 2016 (Table 1).

**Table 1:** Clinical characteristics in summary

No	Age/sex	Symptoms/signs	Localization	Tumor size (cm^3^)	Pathology	% of resection	No. of biopsies
1	42/M	Seizure	RF	15.732	AA (WHO III)	100	4
2	71/F	Headache, somnolence	RT/P	126.759	GBM	100	2
3	49/M	Recurrent	RF	26.4	DA (partial AA, WHO III)	100	7
4	62/F	Aphasia, right prosopolegia	LF	68.04	GBM	100	3
5	48/F	Headache, left hemiparesis	LF/T/I	84.48	GBM	100	5
6	66/M	Headache, somnolence	LP/O	122.4	GBM	88.6	6
7	36/F	Headache, IICP	RF/T	28.7	GA (partial AA, WHO III)	100	2
8	50/F	Headache, left hemiparesis, IICP	RF	37.44	GBM	100	2
9	71/F	Left hemiparesis	LF/P	36	GBM	100	5
10	49/M	Seizure	RF	70.119	OD (WHO III)	100	5
11	35/M	Seizure	LF	49.02	OD (WHO III)	100	2
12	49/F	Recurrent	RF/T/I	94.875	rGBM	99.6	4
13	41/F	Headache, aphasia	LF/T/P	81.567	AA (WHO III)	100	3
14	61/F	Seizure	RF/T/I	21.06	DA (partial AA, WHO III)	100	2
15	26/F	Seizure	LF	44	OD (WHO III)	100	4
16	34/F	Recurrent	LF	13.888	rGBM	100	8
17	45/M	Seizure, left hemiparesis	RP	36	AA (WHO III)	100	2
18	32/M	Seizure, right tendon hyperreflexia	LF	13.32	GBM	100	3

*F* female, *M* male, *L* left, *R* right, *T* temporal lobe, *P* parietal lobe, *O* occipital lobe, *I* insular lobe, *GBM* glioblastoma multiforme, *rGBM* reccurent glioblastoma multiforme, *AA* anaplastic astrocytoma, *DA* diffuse astrocytomas, *OD* oligodendrogliomas

### Selected criteria [8]

Patients 1, age ranged between 18 and 75 years; 2, who were newly diagnosed, untreated or have relapsed MG, with certain pathological results after postoperative confirmation; 3, according to the surgery after 24–72 h of intracranial MRI examination; 4, according to the enhancement area of postoperative MRI, the surgeon and the imaging department evaluates the total resection of the tumor.

### Exclusion standard

(1) Patients under 18 years old and more than 75 years old were excluded; (2) tumors originating from the brain stem; (3) excluding patients who are suffering low-level gliomas or non-tumors whose MRI showed enhancement areas; (4) renal insufficiency of patients; (5) patients with hepatic insufficiency; (6) other parts of the body with active malignant tumor patients; (7) preoperative tumor MRI enhancement and postoperative pathology proved to be metastatic tumor patients; (8) according to the operation, a single specimen retention of the case should be excluded.

### Definition description

#### Total resection criteria for surgery and post-operation

Total resection of the glioblastoma during surgery was based on the surgeon and the navigational judgment of the operation. Total resection was according to complete disappearance of the FLS staining of tumor tissues, and complete disappearance of the enhanced tumor tissues under neuronavigated operation. While postoperative total resection according to the postoperative brain enhanced MRI after 24–72 h, less than 0.175 cm^3^ volume of the residual postoperative enhancement was considered as total resection [8, 9]. Some cases of total resection were performed larger than the postoperative-enhanced MRI region; these cases could be called as extended resection or ultra-total resection.

#### Standard for postoperative tumor imaging recurrence

The MRI findings showed that the area of tumor resection was larger than 0.175 cm^3^, which was considered to be recurrent [8, 9].

### The method of developing FLS in operation

Twenty percent fluorescent sodium was obtained from Guangzhou Baiyun Mountain Ming Xing Pharmaceutical Co., Ltd. (National Drug Code: H44023400). Before the use of 20% FLS, diluted to 3%, skin test was performed with 5 ml deep vein injection to observe the patient’s vital signs and rashes and other abnormalities, and then diluted to 1%. The dosage was in accordance with the patient’s weight, i.e., 2–3 mg/kg. Drug delivery time: injected the drug just before skin cut after anesthesia induction was begun. Drug delivery: single dose intravenous injection.

### Procedure control

#### The use of neuronavigation in surgery

This procedure uses the frame-free brainlab neural navigation. Routine brain MRI scans were performed in the 1 week before operation. Neuronavigation was recorded using gadolinium-enhanced T1WI sequence, and a surgical plan was established to enhanced boundaries with T1WI. The boundary of neuronavigation was not used as a major criterion for complete resection of tumors. For central sulcus MG, the scanning of diffused tensor imaging (DTI) was used to assess the adjacent relationship between tumor and subcortical fibrous bundles.

#### Combined use of electrophysiological monitoring in operation [10]

MG in the central sulcus area maximize safe resection of tumors while ensuring the integrity of the patient’s movement and sensory function. During the operation, the neural electrophysiological monitoring was performed to complete the resection of the tumor while protecting the vital nerve function as intact as possible.

#### Tumor resection during surgery

After exposing the tumor tissue, the Pentero 900 microscope was used to adjust the filter to the YELLOW 560 nm mode. Most of the time, the surgeon can remove the tumor tissue in YELLOW 560 nm mode, and it is convenient in the YELLOW 560 nm mode to be converted to white light mode by switching the button when it required to stop bleeding or to obtain a pathological specimen. During the operation, the surgeon uses the suction device to absorb the blood from the field of vision as far as possible in order to avoid the situation that leads to blurred vision. Sometimes, in order to avoid damage to the normal brain tissue, ultrasound absorbers from the internal and external absorption of tumor tissues were used until the fluorescence staining of the tumor tissue is completely removed.

### Retention of pathological specimens

During the operation, under the real-time YELLOW 560 mode, regardless of tumor location, according to the yellow staining degree of the tissue specimen is labeled as “no yellow dye,” “low yellow dye,” and “bright yellow dye” levels. All tissue specimens were removed and immediately applied with 10% formalin fixation and embedded for pathological analysis. In the tumor boundary area, pathological specimens obtained randomly from each patient were marked as “no,”, “low,” and “bright” yellow according to the yellow fluorescence staining levels.

### Experiment main reagents

GFAP and Ki-67 were purchased from Fuzhou Mai Xin Company.

Sheep anti-rat/rabbit IgG (KIT-5030) were purchased from Fuzhou Mai Xin Company.

### Experimental main equipment

Leica RM2235 type electric paraffin slicer (German Leica Company).

Leica BONDTM Automatic IHC dyeing system (German Leica Company).

### Specimen treatment

All specimens were fixed with 10% formalin, conventional paraffin embedding, slicing machine adjusted to thickness of 4 μm, and sliced continuously. After slicing, HE was stained conventionally and GFAP and Ki67 were stained by immunohistochemistry.

### Immunohistochemical staining (en vision method)

The 4 μm paraffin sections were fixed and washed, and extreme care must be taken to avoid peeling off the sections. The slides were specially repaired by citric acid working fluid (for Ki67) by incubating at 120 °C temperature for 2 min. And GFAP was no need to be repaired.

### Results interpretation and evaluation

GFAP staining of tissue cell cytoplasm of yellow or brown-yellow granules were considered positive, and no color or faint yellowish granules were considered negative.

Compute the Ki-67 labeling index (Ki-67 LI). Ki-67 LI is defined as the percentage of the total number of cells that are Ki-67 positive. For the region of necrosis and vascular endothelial cells, cells can be differentiated when the nucleus that is less than 2 mm is not counted. In view of less than 1 cell per low magnification, LI is considered to be less than 0.1% , is not visible under the microscope, and is calculated as 0 [4].

### Postoperative follow-up

The follow-up of outpatients, inpatients was done through cell phone, SMS, interview, WeChat, Tencent QQ, and email. Imaging evaluations were performed at 1 month, 3 months after surgery, and every 6 months. All patients underwent MRI scanning and contrast-enhancement (CE) of the brain in 24–72 h after operation, 1 month after operation, 3 months, and every 6 months. The associated complications were observed from elevated blood pressure, seizures, tracheal spasms, or allergic reactions following the injection of FLS.

### Statistical analysis

Continuous variables are described in average, median, and standard deviation. SPSS19.0 statistical software was used and *χ*^*2*^ test with multiple composition ratios were used. *p* < 0.05 was considered statistically different.

## Results

### General information results

The patients included in this study were 18 cases, including 7 male cases and 11 female cases. The average age was 48.2 years (26–71 years old). Main symptoms and postoperative pathological classification had been listed in Table 1.

### Fluorescence imaging results

**Fig. 1:**
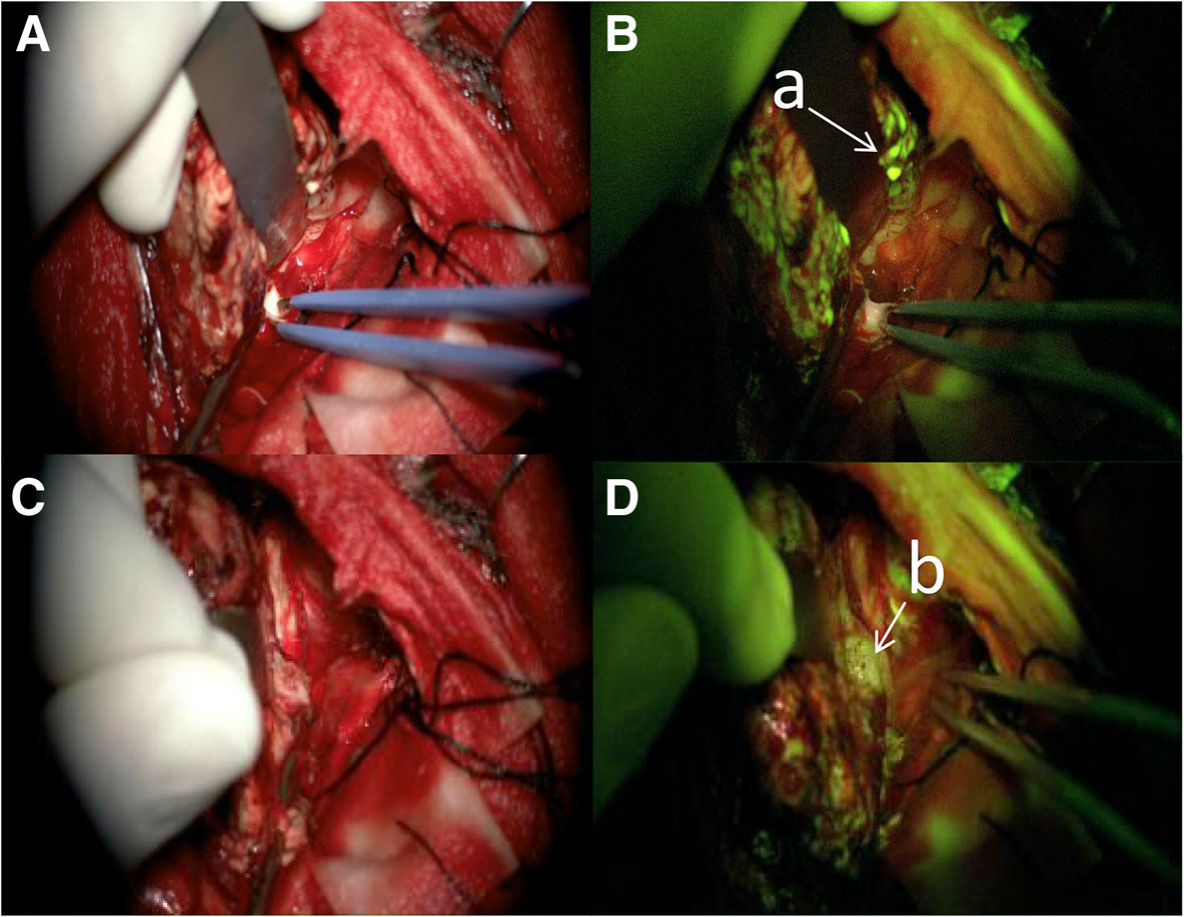
Case 16. Comparison between white light and YELLOW 560 nm mode under Pentero 900 microscope was developed. **a**, **c** For white light, the boundary display was not clear, especially the direction of the bipolar, and difficult to distinguish. **b**, **d** Two figures showed the YELLOW 560 nm mode of development, where the dyed bright fluorescent color of the tumor tissue and the surrounding non-fluorescent tissue boundaries were clear, facilitating the removal of tumors. In **b**, “a” is the “light” fluorescent color, and the bipolar refers to the “none” fluorescent color. In **d**, “b” refers to a “low” fluorescent color and can be easily differentiated from the “none” fluorescent color tissue (indicated by bipolar in **b**)

**Fig. 2:**
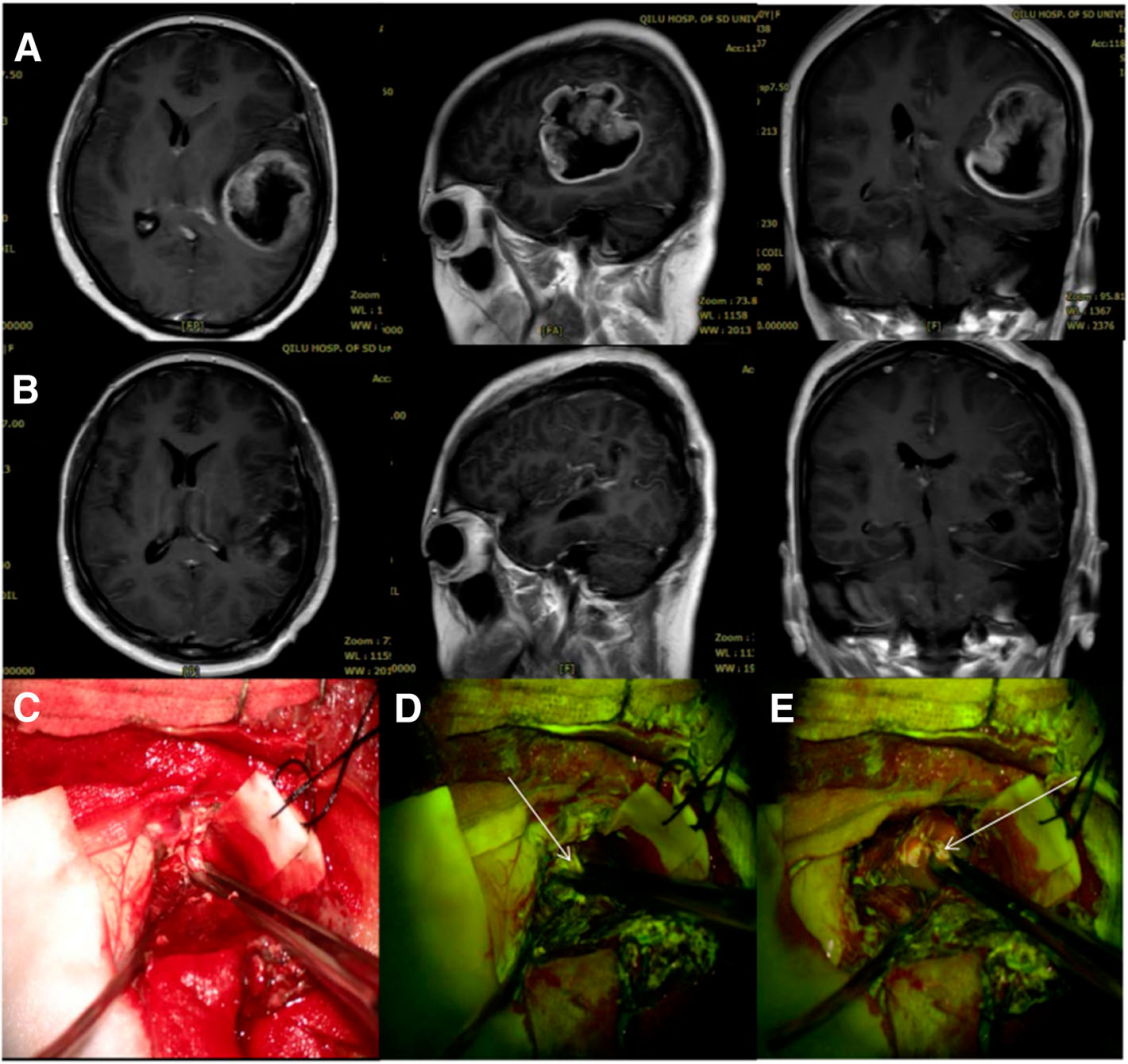
Case 5. **a** T1WI enhancement MRI of the left frontal temporal insular lobe. **b** Postoperative MRI in 24 h. **c** Under Pentero 900, white light showed the tumor boundary, the boundary was unclear, and cannot be distinguished. **d**, **e** Tumor boundaries under YELLOW 560 nm. Fluorescence and non-fluorescent tissues are easily differentiated under the YELLOW 560 mode. **d** Specimens obtained in the boundary of “low” fluorescence color (white arrow). **e** “None” fluorescence to obtain specimen (white arrow)

One percent FLS was intravenously injected before skin incision after induction of general anesthesia. The first 17 cases were administered with 3 mg/kg weight of FLS, followed by 2 mg/kg weight, and both obtained the same effect (see Fig. 1 and Fig. 2). After opening the dura, i.e., intravenous FLS injection for 20–40 min, it gathers tumor tissue without spilling into normal brain tissue. After craniotomy, all tumors were stained by FLS in YELLOW 560 nm mode. The tumor tissue showed a bright fluorescent color (see Fig. 1b (a)), especially in the MRI-enhanced area. But in the necrotic region of the tumor or in the area where the MRI was not enhanced, it showed a “low” or “none” fluorescent staining (see Fig. 1d (b)). The liquid region of cystic tumors can still be shown as bright fluorescence in color and obviously lighter than that in the cerebrospinal fluid. Even at very low concentrations of FLS (2 mg/kg), the YELLOW 560 nm model of the Pentero 900 microscope can easily differentiate between fluorescent and non-fluorescent tissues.

### Surgical results

**Fig. 3:**
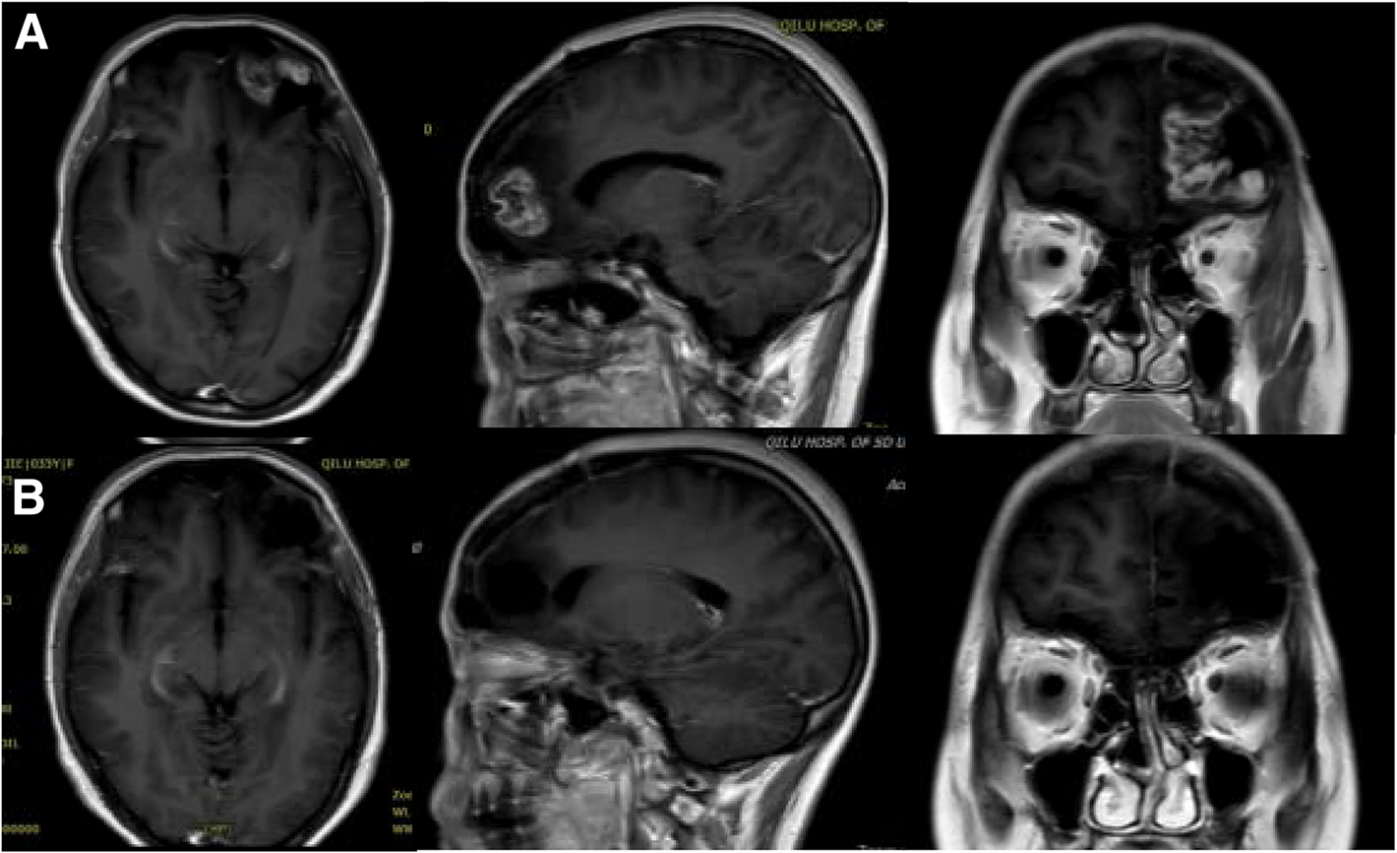
Case 16. Performed on the axial, sagittal, coronal, and enhanced MRI. **a** Imaging the postoperative recurrence of the left frontal GBM after first operation for 2 years, and the volume was 13.9 cm^3^. **b** In 72 h, the axial, sagittal, coronal postoperative-enhanced MRI imaging showed no enhanced area residue

The average tumor volume was 53.88 cm^3^ (13.32–126.759 cm^3^). The total resection was performed in 16 cases (see Fig. 3 and Fig. 2). The 69 pathological specimens were randomly obtained from 18 cases (32 bright-, 18 low-, 19 non-fluorescence). The postoperative KPS score was slightly higher than before, though no statistically significant difference (average preoperative 82 vs. postoperative 83, *p* = 0.566). Five patients reported a short KPS score reduction (cases 4, 6, 8, 13, and 17) after surgery. In addition, eighth case had permanent mild hemiplegia, with short-term recovery to the preoperative state. No venous thrombosis and pulmonary embolism occurred after operation. The color of the skin, mucus membranes, and urine of the patient after being injected with FLS was yellow and disappeared after about 24 h after the operation. No related serious adverse events were found.

Two patients (case 1 and 18) had epileptic seizures in the early postoperative period (1 month), considering the preoperative state of the patients.

### Follow-up results

Sixteen cases were completely followed up (cases 4 and 11 were lost), with a follow-up rate of 88.9% (16/18). Complete radiotherapy and chemotherapy (Stupp regimen) were completed in 10 cases (10/18).3 patients died of tumor progression (cases 5, 6, and 8) and 1 patient died of severe pneumonia.

### Pathological examination results

#### Relationship between the expression of GFAP and fluorescence levels of MG (Table 2, Fig. 4, Fig. 5)

In 69 specimens, 61 were GFAP positive. SPSS 19.0 Statistical software was used to compare the relationship between the positive rate of GFAP expression and the levels of fluorescence of MG, and *χ*^*2*^ test with multiple composition ratios was used, revealing no statistically significant difference (*χ*^*2*^ = 0.627, *p* = 0.731).

**Table 2:** Relationship between the expression of GFAP, WHO grades, and fluorescence levels of MG

Items	Fluorescence levels			Degree of freedom	*χ* ^*2*^	*p*
	None	Low	Bright			
GFAP (+)	17	15	29	2	0.627	0.731
GFAP (−)	2	3	3			
WHO III	12	7	12	2	3.531	0.171
WHO IV	7	11	20

**Fig. 4:**
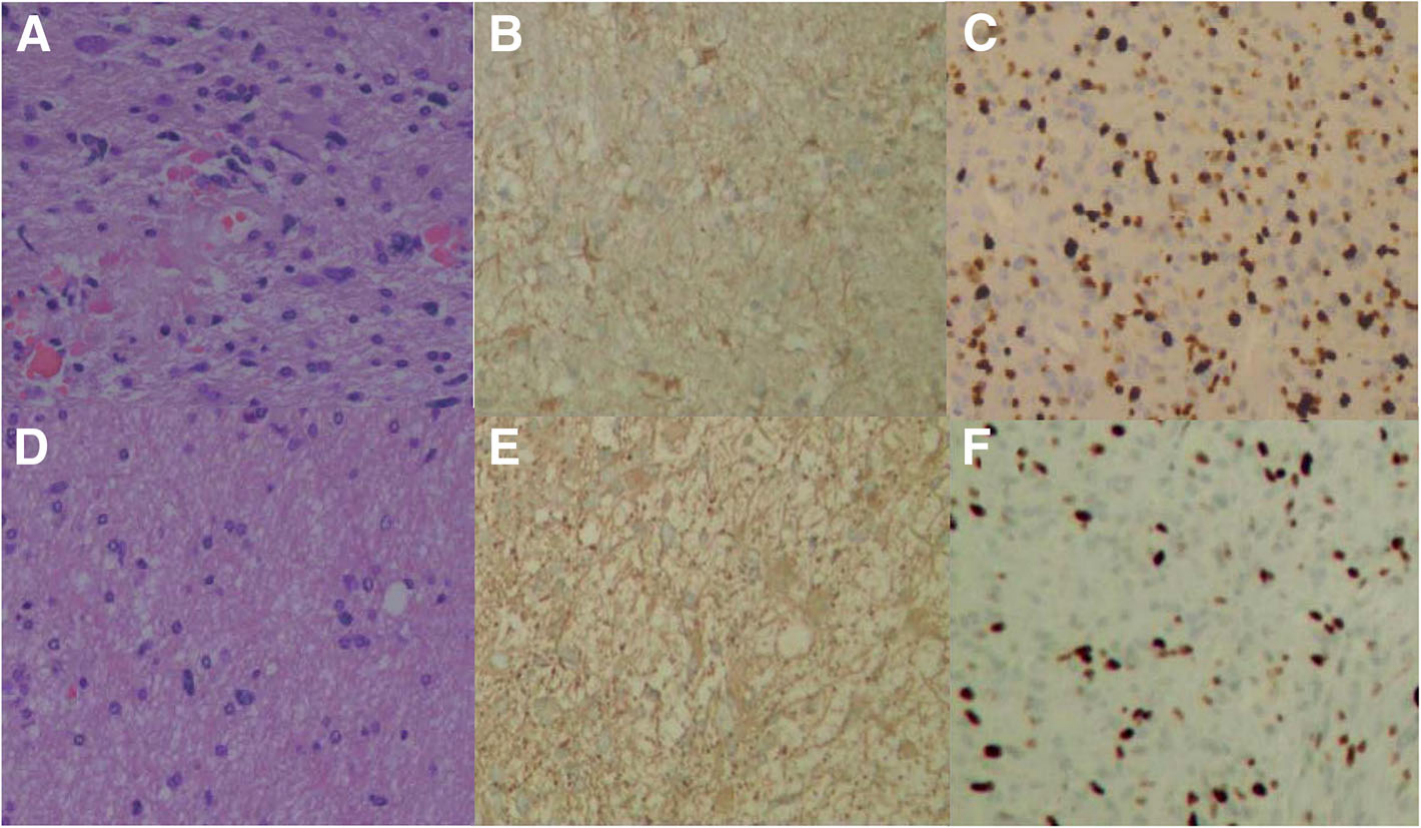
Case16. **a**–**c** The biopsies obtained from Fig. 1b (a), stained by HE X200, GFAP X200, and Ki-67 X200, respectively. **d**–**f** Biopsies obtained from Fig. 1d (b), stained by HE X200, GFAP X200, and Ki-67 X200, respectively. **a** GBM cell nuclear split pleomorphism and was accompanied by a large number of vascular distributions. **b** Lighter color with GFAP staining, which was caused by lesser cytoplasm. **c** Ki-67 LI was 45%. **d** Brain tissue infiltration by tumor cells, nuclear atypia was uncommon as shown in **a**. **e** Deeper color with GFAP staining, which was caused by full cytoplasm. **f** Ki-67 LI was 25%

**Fig. 5:**
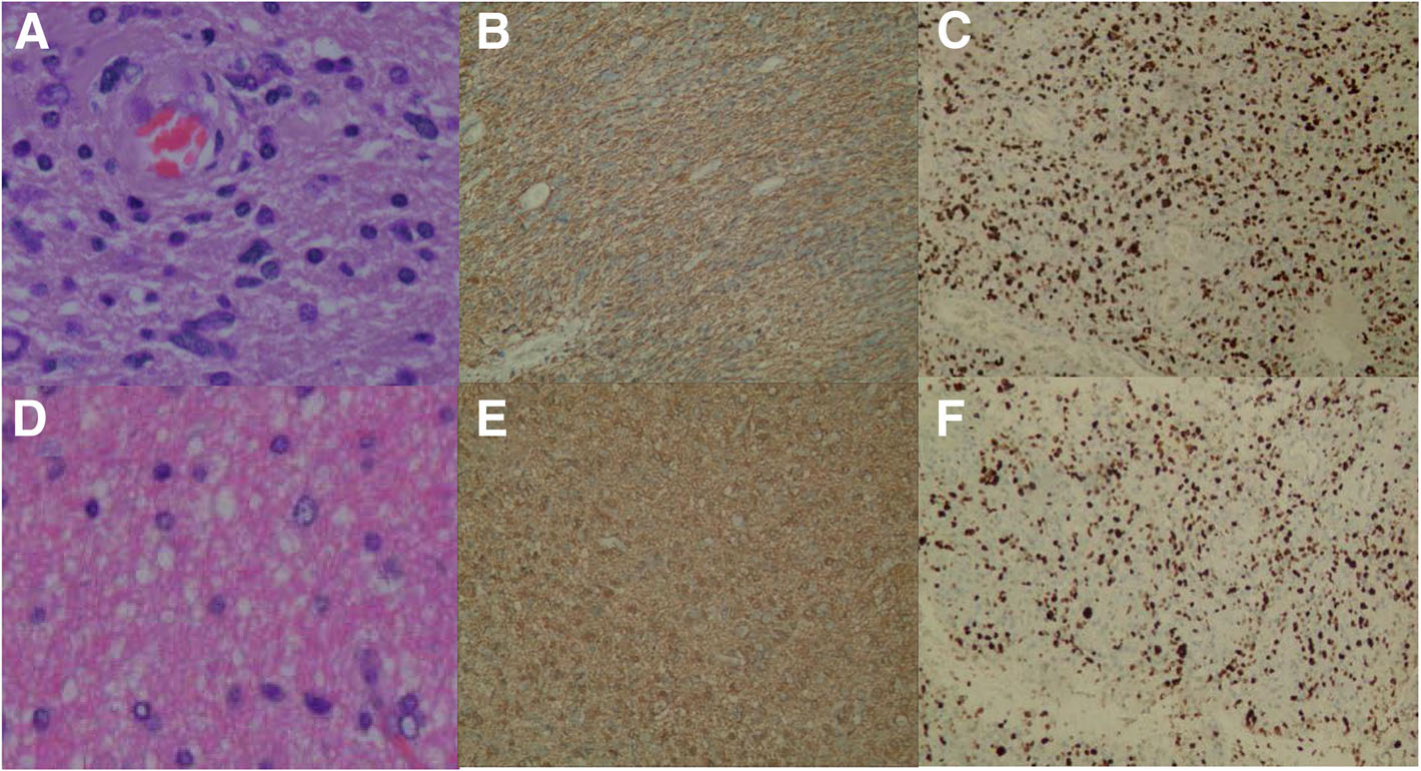
Case 5. **a**–**c** Observed HE staining under × 400, GFAP staining under × 200, and Ki-67 staining under × 100 at the specimens obtained from Fig. 2d (white arrow). **d**–**f** Observed under × 400 of HE staining, under × 200 of GFAP staining, and under × 100 of Ki-67 staining at the specimens obtained from Fig. 2e (white arrow). From **a**, the nuclear split pleomorphism of GBM tumor are seen obviously and accompanied by vascular distribution. The GFAP staining in **b** showed lighter color than **e** caused by less cytoplasm in **b**. **c** Ki-67 LI 50%. **d** Figure contains tumor cells infiltration; abnormity was uncommon. **e** Glial cells were plump, and GFAP staining was darker. **f** Ki-67 LI was 30%

#### The relationship between WHO grading and fluorescence levels (Table 2)

Comparing the relationship between the WHO grading and fluorescence levels of MG revealed no statistically significant difference (*χ*^*2*^ = 3.531, *p* = 0.171).

#### Ki-67 LI relation to fluorescence levels (Table 3, Fig. 4, Fig. 5)

Comparing the relationship between Ki-67 LI and fluorescence levels of MG revealed statistically significant difference (*χ2* = 14.678, *p* = 0.0050.014).

**Table 3:** Relationship between the expression of GFAP, WHO grades and fluorescence levels of MG

Ki-67 LI %	Fluorescence degree			Degree of freedom	*χ* ^*2*^	*p*
	None	Low	Bright			
< 20	7	3	2	4	14.678	0.005
20–40	9	8	9			
≧ 40	3	7	21			

## Discussion

### Effect of FLS navigation on the removal rate of MG

Nowadays, there were three main fluorescent agents used for guided resection of glioblastoma including indocyanine Green (ICG), FLS, and 5-ALA. The removal of MG by the ICG-guided resection involves a short time, which could be helpfully used to obtain specimens but little useful for continuous navigation monitoring. ICG navigation combined with other navigations (such as 5-ALA) was mainly used for examination of residual tumors involving a short time after resection of MG [11]. 5-ALA had not yet been approved by the Committee on Food and Drug Administration which is an endogenous luminescent agent, with optical instability, partly low sensitivity, and specificity. Because of its “light bleaching,” the tumor edge was not clearly displayed [12]. 5-ALA also had the disadvantages of photo-induced toxicity, expensive equipment, and complicated application process. Hence, 5-ALA had much difficulty in clinical popularization. FLS in ophthalmic fundus angiography had been safely and effectively used for many years, and was used to identify tumors in neurosurgery [13, 14]. Shinoda et al. [15] reported resection of 32 glioblastomas, and the total resection rate reached 84.4%. Chen B et al. [16] reported 10 cases of MG with complete resection of 80%, while only 33.3% of MG in control group (12 cases). KOC [17] resected 47 cases of glioblastomas demonstrating a total resection of 83% while control group (33 cases) showed 55%. Though there was no significant difference of influencing the lifetime (43.9 weeks and 41.8 weeks). 20 mg/kg FLS obtained clear imaging, and 10 cases of glioblastoma were expected to be resected. Kuroiwa et al. [18] after using special filter, 10 cases of glioblastoma were resected by intravenous injection of 8 mg/kg FLS. F. Acerbi et al. [8] using Pentero microscope intravenously injected 5 mg/kg FLS, then 12 cases of GBM obtained a total resection of 75%, and the remaining patients with tumor resection volume of 90.5% (82.6–99.9%). In 2013, K.M. Schebesch et al. [7] reported that under the YELLOW 560 nm filter, with intravenous injection of 200mg FLS (3-4mg/kg), 35 cases of intracranial tumors, including 22 cases of MG, were resected. In our preliminary study, the total resection rate of MG under the guidance of FLS was 92.1%. The 6 month-PFS (92.3%) and median survival period (11 months) after the resection of glioblastoma were similar to those of FLUGLIO [8, 19]. Of the 18 cases, the total resection was achieved in 16 cases with multiple regional retentions of specimens according to the postoperative-enhanced MRI. Under the white light mode, the tumor tissue was blurring in the boundary between the tumor and the surrounding tissues. While under YELLOW 560 nm mode, the normal tissue and the tumoral tissue could be clearly identified (Figs. 1 and 2). Postoperative KPS score decreased before operation, and a slight increase after the operation, though the difference was not statistically significant (average preoperative 82 vs. postoperative 83, *p* = 0.566). No postoperative complication was associated with FLS, and thus it was considered safe and effective for the removal of MG by FLS navigation.

### The relationship between GFAP expression and the levels of FLS in MG

GFAP, an acidic protein belonging to the family of intermediate silk proteins, is the cytoskeleton protein of specific astrocytes [20], which was first separated from the white matter plaque of multiple sclerosis patients [21]. The function of nutritional support neurons plays an important role in shaping and maintaining normal morphology of glial cells [20, 22]. GFAP is normally expressed in astrocytes, ventricular membrane cells, and so on, and is considered as the iconic protein of astrocytes [23]. GFAP-positive expression could be found in astrocytoma, ventricular duct tumor, mixed glioma of the astrocytes line, giant cell astrocytoma, pleomorphic yellow astrocytoma, astrocytoma, glioma sarcoma, and so on [24]. As a reliable protein marker, it was widely used in the clinical immunohistochemical identification of glioma and non-gliomas [25]. In this group, 61 specimens of GFAP were positive. There was no correlation between the expression rate of GFAP and fluorescence levels (Fig. 4b, e and Fig. 5b, e). The result might be due to that (1) the normal glial cells also express GFAP, and our study observed that the same patient had different fluorescence level regions and obtained the different specimens when the color of fluorescence was darker (Fig. 1b (a) vs. Fig. 1d (b), and Fig. 2d white arrow vs. Fig. 2e white arrow) while the color of staining was lighter (Fig. 4b vs. Fig. 4e, and Fig. 5b vs. Fig. 5e). According to the literature, with the increase of the malignant degree of astrocytoma, the production of GFAP decreased [26]. Immunohistochemistry showed that the GFAP-positive staining rate and staining color of MG were lighter than those of normal brain tissue. We had confirmed this discipline through only using semi-quantitative analysis. (2) Semi-quantitative analysis has a certain degree of bias. It may be more instructive to study the quantitative expression of GFAP in the future. (3) Due to multiple-sources of MG, the positive rate of molecular pathology expression also changed a lot.

### Relationship between WHO grading and fluorescence level in MG

The higher the malignant degree of glioma, the worst will be the prognosis, and the higher will be the WHO grading. We supposed that glioma with different malignant degrees might have influence on the developing intensities of FLS. But in this group, there was no significant correlation between the WHO grading and fluorescence levels (*χ2* = 3.531, *p* = 0.171). The reason may be due to (1) after injection, FLS transmitted through the destruction of the blood-brain barrier and agglomerated in the extracellular matrix. Diaz and others reported that [27] FLS showed obvious development in gadolinium-enhanced preoperative MRI area. It indicated that the development of FLS was similar to the enhancement mechanism of gadolinium injection, which was related to the damage of blood-brain barrier. However, some studies, including our early observation combined with neuronavigation, found that there was still FLS [28] outside the gadolinium-sprayed amine-enhanced range. So, there are still many unsolved reasons in the distribution mechanism of FLS. (2) This small sample size was not enough to show the relationship between WHO grading and fluorescence levels. (3) WHO grading gradually developed to molecular pathology; many molecular pathology mechanisms are unclear, and different types of MG characteristics according to the molecular pathological diagnosis may be different.

### Relationship between Ki-67 and fluorescence levels in MG

Ki-67, a nuclear antigen of proliferating cells, can be detected in the nuclear plasma and mitotic phase of the cell transferring to the chromosome surface. In MG, Ki-67 acts as a cell proliferation marker that was more specific than PCNA [22]. The percentage of Ki-67-positive expression can reflect the degree of malignancy [29]. Ki-67 was expressed in all levels of astrocytoma and showed high expression levels in the higher grades of tumors [30]. Kiss et al. [31] observed that low- and very low-density Ki-67 LI had longer survival rates. Torp et al. [32] also reported that Ki-67 LI was associated with malignancy of astrocytoma, with high levels of Ki-67 LI than with low levels of LI, exhibiting a worse prognosis. Bouvier et al. believed that greater than 5% of Ki-67 LI was considered as a risk factor for tumor progression and poor prognosis [32]. In this group, all the patients showed Ki-67 expression and was graded by Ki-67 proliferation index LI. Ki-67 LI was correlated with fluorescence levels, and the difference was statistically significant (*χ2* = 14.678, *p* = 0.005). This suggested that the removal of MG through FLS navigation can reduce the MG cells and reduce Ki-67 LI, improving the prognosis of patients (Fig. 4 and Fig. 5). Bouvier and other studies have also found that Ki-67 Li may not be an independent risk factor, but low levels of Ki-67 and total resection were achieved, and continuous postoperative chemotherapy patients had a better prognosis.

Although the expression of Ki-67 was still observed (Fig. 4f and Fig. 5f) in the near-positive boundary biopsy of FLS staining (Fig. 1d (b) and Fig. 2e white arrow), it had been significantly reduced after surgical resection (*χ2* = 14.678, *p* = 0.005). Furthermore, it has been found in our earlier study that there was still fluorescein dye outside the area of MRI enhancement according to preoperative MRI registered by neuronavigation [19]. Thus, the fluorescein-stained regions could be included and larger than the contrast-enhanced regions. More aggressive or super-total resection of MGs could be deemed to the reasonable treatment strategy for MGs [5]. For the development of FLS and to help in glioma resection, more clinical data should be studied and discussed.

## Conclusion

FLS navigation was helpful in resecting the MG. In the boundary area, fluorescence levels and GFAP-positive rate, WHO classification level were not correlated. The development of FLS may be related to the destruction of blood-brain barrier, but the mechanism of distribution of FLS still needs further study and discussion. Fluorescence levels and Ki-67 proliferation index has correlation. It is suggested that fluorescein-sodium-guided resection of MG can achieve the function of “reducing tumor cell,” which reduces the proliferation index in the lesion area. This subsequently provides better basis for postoperative radiotherapy and chemotherapy, and hope to reduce recurrence and improve prognosis.
